# Epstein–Barr virus and multiple sclerosis: lesson learned to develop better nonhuman primate models

**DOI:** 10.1038/s12276-025-01482-5

**Published:** 2025-06-30

**Authors:** Hai Duc Nguyen, Daesik Kim, Yong-Hee Kim, Erik Flemington, Gavin Giovannoni, Chung-Gyu Park, Woong-Ki Kim

**Affiliations:** 1 Division of Microbiology, Tulane National Primate Research Center, Tulane University, Covington, LA USA; 2 Transplantation Research Institute, Seoul National University, Seoul, South Korea; 3 Department of Pathology and Laboratory Medicine, Tulane University School of Medicine, New Orleans, LA USA; 4 Blizard Institute, Faculty of Medicine and Dentistry, Queen Mary University of London, London, UK; 5 Department of Microbiology and Immunology, Seoul National University College of Medicine, Seoul, South Korea; 6 Department of Microbiology and Immunology, Tulane University School of Medicine, New Orleans, LA USA

**Keywords:** Autoimmunity, Autoimmune diseases

## Abstract

Multiple sclerosis (MS) is a chronic autoimmune disorder with a complex etiology, and Epstein–Barr virus (EBV) is considered the leading cause. While understanding the role of EBV infection in the pathogenesis of MS in human subjects is crucial, animal models, particularly nonhuman primates (NHPs), would provide an ideal controlled environment for testing EBV hypotheses and identifying potential therapeutic targets. Here in this Review we address clinically relevant questions regarding the link between EBV infection and MS to inform the development and refinement of virally induced NHP models. We focus on integrating known EBV-related risk factors for MS, including age at infection, infectious mononucleosis, genetic predispositions such as the human leukocyte antigen (HLA)-DR15 haplotype, sex-specific susceptibility, low vitamin D levels and CD8^+^ T cell deficiency. We also explore the application of these risk factors in model development, investigate why most EBV-infected individuals do not develop MS and propose potential disease-modifying therapeutic options and vaccines. Integrating these approaches into NHP models will improve our understanding of MS pathogenesis and guide the development of targeted strategies for disease management and prevention. We propose to develop a refined EBV infection NHP model of MS coupled with CD8^+^ cell depletion and other inclusion and exclusion criteria.

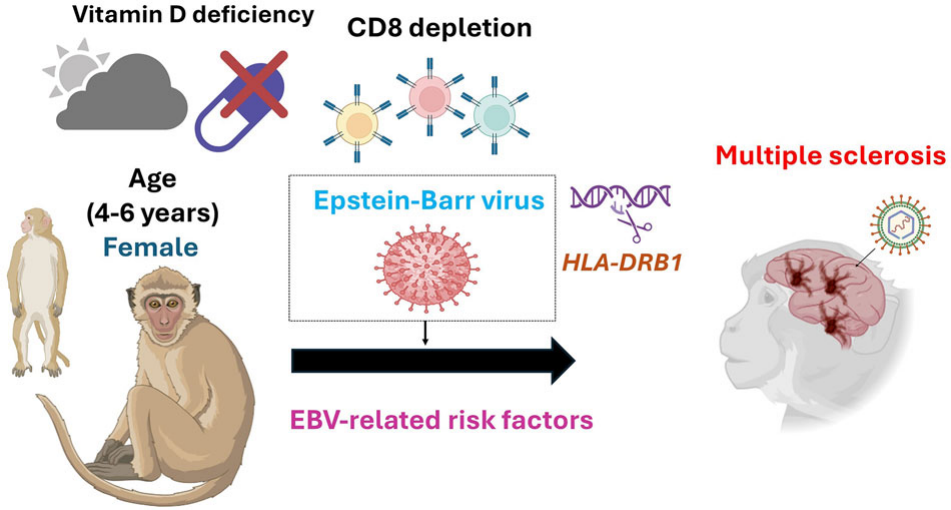

## Linking Epstein–Barr virus to multiple sclerosis pathogenesis

Multiple sclerosis (MS) is a disease in which the immune system attacks the brain and spinal cord. Scientists believe that a virus called Epstein–Barr virus (EBV) might play a role in causing MS. Researchers looked at how EBV-related risk factors of MS, both genetic and environmental, impair CD8^+^ T cell responses and might contribute to MS. The Review proposes to use nonhuman primate models, such as monkeys, to better understand these interactions because they are like humans in many ways. The Review suggests that infection with EBV-related monkey EBV, combined with experimental CD8^+^ cell depletion and other factors to consider such as age at infection, may increase the frequency of developing MS-like diseases in monkeys. The researchers concluded that nonhuman primate models can be used to help test new treatments and understand why some people with EBV do not develop MS.

This summary was initially drafted using artificial intelligence, then revised and fact-checked by the author.

## Introduction

Multiple sclerosis (MS) is a chronic inflammatory disease characterized by inflammation, demyelination and varying degrees of neuroaxonal loss in the central nervous system (CNS)^1^. Demyelination results in a broad spectrum of neurological symptoms, including motor and sensory deficits and cognitive and visual impairment. The development of MS is complex and seems to involve a combination of environmental, genetic and immunological factors^2^. Environmental factors, such as a Western diet, vitamin D deficiency, smoking and disrupted microbiota, may interact with certain genetic variations linked to MS, resulting in dysregulated immune responses^3^. A recent epidemiological study found a correlation between infection with the Epstein–Barr virus (EBV) and an increased susceptibility to developing MS^4^. EBV, a ubiquitous human herpesvirus, is known to establish lifelong latent infection in B lymphocytes and has been implicated in numerous diseases, including MS. Although the exact etiology of MS remains elusive, accumulating evidence suggests that EBV infection plays a pivotal role in its pathogenesis^3^. Almost all patients with MS test positive for EBV antibodies, and those who experience symptomatic primary EBV infections, such as infectious mononucleosis, are at a considerably higher risk of developing MS later in life^3^. The relationship between EBV and MS is complex and influenced by several factors, including the timing of EBV infection, genetic predisposition and the immune response. For instance, the risk of developing MS is notably higher when EBV infection occurs during adolescence or adulthood compared with early childhood^2,5^. Sex differences, genetic factors such as specific human leukocyte antigen (HLA) haplotypes (for example, HLA-DR15) and environmental factors (for example, vitamin D levels) also influence susceptibility to MS. Despite this knowledge, the mechanisms through which EBV contributes to MS remain poorly understood, particularly in the context of age and CD8^+^ T cell function. Nonhuman primate (NHP) models offer a unique opportunity to bridge this knowledge gap^6^. NHPs, particularly rhesus macaques, are valuable for studying the complex interactions between EBV and MS owing to their physiological and immunological similarities to humans. These models would allow researchers to investigate the pathophysiological processes underlying EBV-associated MS and to test therapeutic interventions in a controlled environment^7^. However, current NHP models of MS are limited in their ability to fully recapitulate the disease’s complexity and determine how it relates to EBV. This Review aims to (1) explore EBV-related risk factors for MS, such as the timing of infection, genetic predisposition (for example, HLA-DRB1*15:01), environmental influences (for example, vitamin D levels) and CD8^+^ T cell response; and (2) to develop and refine virus-induced NHP models of MS. This Review also provides a comprehensive overview of how NHP models can be optimized to explore the intricate relationship between EBV and MS, offering new avenues for therapeutic interventions and improving our understanding of MS pathogenesis in the context of EBV infection.

## EBV-related risk factors for MS

Understanding the risk factors that link EBV to MS is crucial to unraveling the complex relationship between viral infections and autoimmune diseases. EBV, a ubiquitous human herpesvirus, is strongly associated with MS, yet not all individuals infected with EBV develop the disease^3^. Identifying the risk factors that affect this relationship can help to refine our understanding of MS pathogenesis and inform the development of targeted preventive and therapeutic strategies. In this section, we will focus on primary EBV-related risk factors that contribute to the progression of MS, including infectious mononucleosis, incubation period, genetic factors, sex differences, vitamin D deficiency and CD8^+^ T cell deficiency.

### Infectious mononucleosis

One of the key EBV-related risk factors for MS is infectious mononucleosis, a common consequence of EBV infection^8^. Infectious mononucleosis is characterized by fever, sore throat, lymphadenopathy and fatigue^9^. It usually arises during primary EBV infection, typically in adolescents or young adults^10^. Mononucleosis and MS are more likely to occur when primary EBV infection occurs after age 10, a period characterized by a reduction in thymic negative selection of autoreactive T cells and a peak in T helper 1 (Th1) cell-mediated immune responses^3^. The association between infectious mononucleosis and an increased risk of developing MS has been well documented in prospective studies^11^: The risk of developing MS in individuals with EBV infection is 32-fold higher compared with uninfected individuals. This risk is even higher when EBV infection leads to symptomatic-to-severe infectious mononucleosis, particularly for those carrying the HLA-DR15 haplotype^12^. Severe infectious mononucleosis might exacerbate immune dysregulation and contribute to the autoimmune response observed in MS^13^. The mechanisms underlying this increased risk may involve higher and more prolonged viral loads and immune activation during acute EBV infection, leading to a cascade of inflammatory events that predispose individuals to autoimmune diseases^3^. EBV also creates an extralymphatic viral reservoir in the CNS during infectious mononucleosis. During this period, approximately 50% of memory B cells may test positive for EBV^14^. In summary, infectious mononucleosis is a key factor in EBV-induced MS and should be carefully considered in NHP model development. Refining these models will improve our understanding of MS and inform targeted prevention and treatment strategies. Integrating these insights into research and clinical practice will improve MS management and patient outcomes.

### Age of infection

Age at initial EBV infection also plays a vital role in the pathogenesis of MS. Primary EBV infection occurring before 5 years of age is frequently asymptomatic, whereas infection during adolescence can lead to infectious mononucleosis, which causes a substantial expansion of atypical CD8^+^ T cells and NK cells. Most individuals maintain effective long-term immune control over the virus with sporadic, rapidly suppressed reactivation events^3^. The majority of children in developing countries acquire asymptomatic EBV infection before age 3 and develop antibodies against EBV by age 10 (ref. ^15^). Early life exposure to microbes including EBV due to unsanitary conditions and poor hygiene in developing countries may contribute to protection against autoimmunity^16^. In developed countries, around 50% of children lack antibodies against EBV by age 10. Of these, many acquire EBV infections orally during their teenage years or early adulthood^10^, around half of which present as acute infectious mononucleosis^17^. These delayed initial infections may explain the increased risk of MS in individuals with a history of infectious mononucleosis^8^: The risk of developing MS is markedly higher if EBV infection occurs during adolescence or early adulthood compared with early childhood^4^. This differential risk may be related to the developmental state of the immune system at the time of infection and the potential for critical immunological disturbances during later stages of life^18^. NHP models of MS must carefully consider the timing of EBV infection for accurate recapitulation of human disease.

### Major histocompatibility complex haplotypes

Genetic predisposition plays a crucial role in susceptibility to MS. One of the most well-established genetic risk factors for MS is the presence of a specific major histocompatibility complex (MHC), the HLA-DR15 haplotype^19^, which interacts with EBV infection to confer a threefold or higher risk of developing MS^20^. HLA-DR15 alleles, including HLA-DRB1*15:01, are known to mediate EBV entry into B cells, raising the possibility that the pathways facilitating viral entry could also play a role in increasing MS risk^3^. The HLA-DRB1*15:01 allele is thought to influence the immune response to EBV, possibly by increasing autoimmune reaction against myelin. In humanized mouse models, the MHC class II molecule HLA-DRB1*15:01 is associated with less effective control of EBV infection^12^, and CD4^+^ T cells activated during EBV infection recognize EBV-transformed B cells in an HLA-DRB1*15:01-restricted manner, exhibiting a higher propensity for cross-reactivity with myelin autoantigens. This cross-reactivity is believed to contribute to the autoimmune processes underlying MS^21^. HLA-DR15 synergistically promotes the activation of hyperreactive T cell compartments that show reduced efficiency in controlling viral infections and harbor cross-reactive CD4^+^ T cell clones^12^. Patients with MS with HLA-DRB1*15 or HLA-B*07 alleles have elevated EBV viral loads, while those with HLA-A*02 have lower viral loads^22^, suggesting that both class I and class II MHC molecules may influence EBV latency control. These associations highlight the role of CD8^+^ T cells, restricted by MHC class I molecules such as HLA-A*02 and HLA-B*07, in differentially mediating antiviral immunity. Effective CD8^+^ T cell responses are crucial for controlling EBV-infected B cells, and impaired CD8^+^ T cell surveillance may contribute to the persistence of EBV and the increased risk of MS. MS-associated risk alleles are linked with altered gene expression patterns, and elevated expression of these HLA alleles can influence peptide selection and presentation, which may contribute to peptide mimicry and autoreactivity^23^. Understanding the interplay between genetic predisposition and EBV infection is crucial for MS research. Studying MHC haplotypes and other risk genes in NHP models, including genetic modifications such as CRISPR–Cas9, can refine MS models and uncover disease mechanisms.

### Sex differences

Sex differences are a well-recognized factor in MS epidemiology and risk^24^. Women are more likely to experience symptomatic EBV infections such as infectious mononucleosis and are subsequently at a higher risk for developing MS compared with men. A study of 2,487 Danish patients with infectious mononucleosis reported that the cumulative risk of developing infectious mononucleosis before age 30 was 13.3% for males and 22.4% for females^25^. A single-center retrospective study found that male patients generally experienced EBV-induced infectious mononucleosis later in life than women, and these infections were more likely to cause headache and leukocytosis^26^. Hormonal differences, immune system variations, oral and gut microbiota, and genetic factors may contribute to this increased susceptibility in women^27^. Estrogen, while known to modulate immune responses—by regulating transcription factor FoxP3, programmed cell death protein 1, interferon gamma (IFN-γ) promoter region, T-bet and cytotoxic T-lymphocyte antigen 4—exerts complex and sometimes opposing effects^28^. The role of estrogen in autoimmunity is highly context dependent^29^. While it can enhance immune activity and contribute to increased susceptibility to autoimmune diseases, it may also exert anti-inflammatory effects in conditions like MS. Notably, MS symptoms often improve during pregnancy, when a shift toward a Th2-dominant immune profile (not Th1) is thought to reduce inflammation and disease activity^30^. The sex differences in MS may also reflect the earlier onset of puberty in females compared with males, and the tendency in heterosexual relationships for the female partner to be younger than the male partner^25^. Females typically have higher numbers of CD4^+^ T cells and a higher CD4^+^/CD8^+^ T cell ratio compared with age-matched males, reflecting a relatively lower proportion of CD8^+^ T cells within the overall T cell compartment^31^. As CD8^+^ T cells are critical for controlling viral infections such as EBV, this lower relative abundance may impair effective viral clearance, potentially contributing to an increased susceptibility to EBV-related conditions such as infectious mononucleosis and MS. Hormonal factors, combined with this immune imbalance, probably contribute to the increased susceptibility of females to MS, where an overactive immune system attacks the nervous system. Incorporating sex-specific differences in EBV infection and MS risk is vital for developing accurate NHP models. Including both sexes or focusing on female animals can clarify how these factors influence disease progression and inform targeted prevention and treatment strategies.

### Vitamin D levels

Vitamin D is recognized for its immunomodulatory properties, particularly its influence on T cell function and the regulation of inflammation^32^. Low levels of vitamin D are associated with an increased risk of MS, probably due to its role in modulating immune system function and influencing the immune response to EBV^33^. The proposed mechanism is that vitamin D increases the availability of CD8^+^ T cells capable of targeting and controlling EBV infection. The vitamin D receptor is expressed at high levels on activated CD8^+^ T cells, suggesting an important role for vitamin D in modulating this activity of CD8^+^ T cells^34^. Vitamin D also enhances the mitogen-induced proliferation of CD8^+^ T cells and lowers the CD4/CD8 ratio in bovine peripheral blood mononuclear cells^35^. Supplemental vitamin D has been associated with increased CD8^+^ T cell count, while vitamin D deficiency correlates with a reduced proportion of CD8^+^ T cells and an elevated CD4/CD8 ratio^36^.

Vitamin D deficiency plays a role in the development of MS by contributing to the depletion of CD8^+^ T cells, which are crucial for controlling EBV infection. Because vitamin D metabolism in NHPs closely resembles that in humans, including hydroxylation pathways (that is, 25-hydroxylation and 1α-hydroxylation) and immunomodulatory effects, NHP models provide a relevant and translationally valuable system for studying the immune consequences of vitamin D deficiency^37^. This hypothesis can be tested in NHP models by examining how vitamin D deficiency impacts CD8^+^ T cell responses, EBV load and MS-like symptoms. Studies using dietary supplementation can clarify the role of vitamin D in immune regulation and EBV control, offering insights into MS pathogenesis and potential therapeutic strategies. Research could also include controlled sunlight exposure to evaluate its effects on CD8^+^ T cell counts, EBV load and clinical symptoms associated with MS.

### CD8^+^ T cell deficiency, EBV control and MS

CD8^+^ T cells play a key role in immune defense against EBV by recognizing viral peptides via TCRs on MHC class I molecules^38^. Upon activation, they induce apoptosis in infected cells through perforin and granzymes while releasing IFN-γ to enhance antiviral immunity and suppress viral replication^39^. A pilot study of ten patients with MS also found elevated levels of interleukin (IL)-4 and IFN-γ mRNA in highly differentiated CD8^+^ T cells^40^. CD8^+^ T cells recognize EBV antigens (for example, EBNA3A, EBNA3B and EBNA3C) essential for the virus’ ability to persist and proliferate^41^. CD8^+^ T lymphocytes specific for the EBV-encoded nuclear antigen 1 (EBNA1)-derived epitope HPVGEADYFEY were also found in the blood and cerebrospinal fluid (CSF) of patients with MS^42^. There was a correlation between antibodies specific to EBNA1 and CD8^+^ T cells that target EBV in individuals with a first neurological episode of MS (called clinically isolated syndrome) and relapsing–remitting MS^43^. Patients with relapsing–remitting MS have high populations of CD8^bright^ cells that specifically recognize the HLA-E–BZLF1 complex. The frequency of CD8^+^ T lymphocytes responding to specific EBV-derived epitopes, including EBNA3A (HLA-A2–CLG) and LMP-2 (HLA-B7–RPP), is higher in patients with MS compared with healthy controls^44^. In EBV infection, CD8^+^ T cells selectively target and eliminate B cells that are infected with EBV^17^. Evidence suggests that multifunctional CD8^+^CD57^+^ cytotoxic T cells in healthy donors could eliminate EBV-infected cells^45^. An analysis of postmortem brain samples from 12 donors with progressive MS and known HLA class I genotype revealed the presence of cytotoxic CD8^+^ T cells that specifically target EBV in the CNS. These CD8^+^ T cells were shown to be positive for CD107a, indicating cytotoxic characteristics, and colocalized to EBV-infected cells^41^. The presence of the EBV lytic protein BZLF1 and the interaction between cytotoxic CD8^+^ T lymphocytes and EBV-infected plasma cells in inflammatory areas in the meninges and white matter have been observed in untreated patients with MS^46^. In individuals with MS, T cell receptor-β sequences of CD8^+^ T cells that react to EBV, including various commonly shared EBV-specific sequences, were shown to be enriched intrathecally.

CD8^+^ T cell deficiency may contribute to the accumulation of EBV-infected B cells in the MS brain^41,47,48^. CNS-recruited CD8^+^ T cells typically provide immune surveillance, but in MS, their reduced frequency and impaired function hinder their ability to control EBV^48–51^. Patients with MS, particularly in advanced stages, show deficits in effector memory CD8^+^ T cells and decreased expression of EOMES and T-bet, key regulators of cytotoxic immunity^47,52–54^. Increased PD-1 expression on CD8^+^ T cells correlates with higher viral loads, suggesting a potential mechanism of EBV-driven MS pathogenesis through CD8^+^ T cell exhaustion^55^. A minority of patients may experience reduced HLA class II expression on B cells, which can prevent the CD8^+^ T cell response to EBV by diminishing CD4^+^ T cell help^56^. However, reports indicate higher frequencies of CD8^+^ T cells that produce IL-4 and IL-10, and higher frequencies of T-bet+ CD4 and CD8 T cells in primary progressive disease^57,58^. Patients with clinically isolated syndrome and those with active MS exhibit a higher percentage of CD8^+^ T cells expressing activation markers CD26 and CD69 compared with patients with inactive disease or healthy individuals^59^. This increased CD8^+^ T cell activity may serve as a compensatory mechanism to control MS progression. IFN-β treatment regulates CD8^+^ T cell activation, potentially influencing disease activity from onset. After IFN-β treatment, the percentage of CD8^+^ T cells expressing IL-10 and IL-13 increases, while those positive for CD26 and CD71 decrease^59^.

**Fig. 1:**
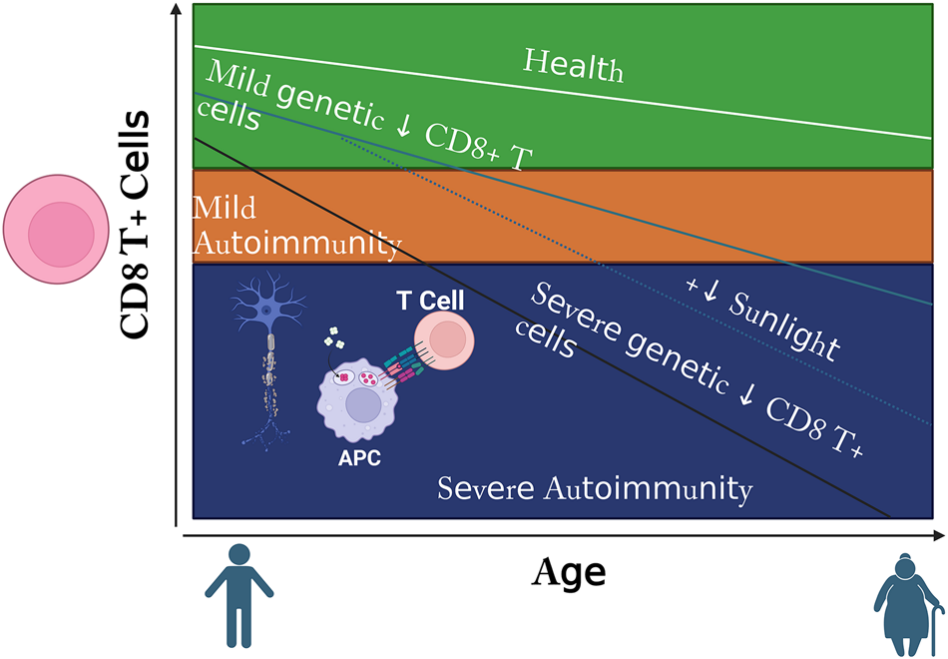
Association between deficiency of CD8^+^ T cells and MS development. In people with a severe genetic CD8^+^ T cell deficiency, autoimmune disorders such as MS appear earlier in life and progress more quickly. Vitamin D and sunlight deprivation at higher latitudes exacerbate genetic CD8^+^ T cell depletion, elevating the risk and development of MS. Adapted from ref. ^17^, license: https://creativecommons.org/licenses/by/3.0/, and modified with BioRender.com.

The total number of CD8^+^ T cells in healthy individuals drops threefold between the ages of 2 and 16 (ref. ^60^). If primary EBV infection occurs during adolescence or adulthood, after the natural decline in CD8^+^ T cells, the same genetic deficiency is more likely to impair EBV infection control^17^, possibly explaining the higher prevalence of MS when the initial infection occurs after puberty^61^. However, a genetically determined CD8^+^ T cell deficiency does not affect the ability to control EBV infection when it occurs in early childhood, unless the deficiency is severe. Aging is related to a decline in immune function, a phenomenon known as immunosenescence, which can affect various immune cells, including CD8^+^ T cells^62^ (Fig. 1). In elderly individuals, CD8^+^ T cells exhibit impaired cytotoxic function, reduced proliferative capacity and altered cytokine production^63^. These changes can impair the ability to control viral infections, including EBV, which leads to the progression of MS.

A case–control study of 64 Australian patients with MS and 68 matched healthy controls found CD8^+^ T cell deficiency in MS, with a reduced response to EBV-infected B cells that worsened with age^64^. This deficiency included fewer lymphoblastoid cell line-specific CD8^+^ T cells, especially in secondary progressive MS, suggesting T cell exhaustion. The age-related decline in CD8^+^ T cells in MS may stem from the depletion of EBV-specific T cells due to prolonged immune activation^64^. The progressive decline of CD8^+^ T cells due to accelerated aging, along with the resulting increase in EBV burden, may be responsible for the age-related accumulation of disability in MS^65^.

Immunosenescence leads to reduced immune cell production, altered cytokine profiles and impaired function^62^. CD8^+^ T cells are particularly affected by aging, including a decline in naive CD8^+^ T cells, diminished proliferative capacity, impaired cytotoxic function and telomere shortening, all of which weaken the response to EBV infection^63,66–69^. Aging significantly affects CD8^+^ T cell function, with profound implications for viral control and the pathogenesis of MS. The decline in CD8^+^ T cell responses in aging due to thymic involution, telomere shortening and altered signaling pathways (PI3K–AKT, mTOR, NF-κB, JAK–STAT and cytokine signaling pathways) may contribute to reduced control of EBV and increased autoimmunity^70–73^.

In summary, CD8^+^ T cells play a key role in MS, especially in controlling EBV infection. Using age-specific NHP models or inducing CD8^+^ T cell depletion can help to uncover their role in MS progression. These models are essential for developing targeted therapies to modulate CD8^+^ T cell responses and manage EBV-driven MS.

## Limitations of current animal models of MS

The experimental autoimmune encephalomyelitis (EAE) model is the most frequently used animal model in MS research, and its historical and scientific importance is well established. Rivers et al. first described it in monkeys in 1933 (refs. ^74–76^). In the 1940s and 1950s, EAE models were developed in mice and rats and established as fundamental tools in MS research^75^. The mouse EAE model has dramatically advanced our understanding of the autoimmune mechanisms and demyelination processes in MS, and its utility extends to mechanistic studies, as it can be induced in various genetically modified mouse strains^77^. The widespread application of this model in MS research is largely due to its amenability to genetic manipulation, capacity for large-scale experimentation and cost-effectiveness^77^.

The mouse EAE model has limitations in replicating the full complexity of human MS, particularly in lesion localization and immune response. While human MS pathology suggests a key role for CD8^+^ T cells and B cells, CD4^+^ T cells dominate in EAE^78^. EAE follows a rapid disease course, whereas human MS often presents with a relapsing–remitting pattern. Thus, while valuable for studying immune mechanisms, the mouse model does not fully reflect the clinical progression of MS^79^. To overcome these limitations, NHP models, particularly the marmoset EAE model, have been developed. The marmoset EAE model exhibits pathological features that more closely resemble human MS and, with a brain structure and immune system more analogous to that of humans, offers greater relevance for studying the intricate pathophysiology of MS^75^. We found changes in CD8^+^ T cell and B cell subpopulations consistent with human MS findings in our studies using the marmoset EAE model; however, marmoset EAE lesions were confined to the spinal cord, with no brain involvement (unpublished data).

### Current NHP models to study MS

NHP models are pivotal in elucidating the complex interactions between EBV and MS and revealing the immunological mechanisms and viral influences that may contribute to MS pathogenesis^6^. NHPs allow a deeper understanding of how EBV influences the risk of developing MS (Table 1) and provide valuable insights into immunological and pathological processes that are difficult to investigate in humans. For example, a rhesus lymphocryptovirus (LCV) model successfully replicated important characteristics of human EBV infection, (transmission through the mouth, CD23^+^ activation, response to EBV antigens and so on)^80^. Monkeys infected with simian immunodeficiency virus develop B cell lymphomas associated with the Epstein–Barr-like cynomolgus B-lymphotropic herpesvirus, which are similar to EBV-associated B cell lymphomas observed in individuals with acquired immunodeficiency syndrome or organ transplants^81^.

**Table 1:** Association between MS and EBV in NHP models.

Authors	Models	Treatment	Key findings	Outcomes	Reference
Haanstra et al.^82^	Rhesus macaques	- Herpesvirus papio (HVP) - Encephalitogenic self-peptide MOG_34–56_ and capsid protein of CMV (CMVmcp_981–1003_)	↑ CD3^+^CD8^+^ T cells ↑ CD8^+^CD56^+^ T cells ↑ CD3^−^CD56^+^ NK cells ↑ perivascular inflammatory lesions (CD3^+^ and CD68^+^ cells)	EBV-induced B-lymphoblastoid cells can cause encephalitis	^82^
Jagessar et al.^83^	NHP B cells	LCV	↑ CD70 and CD80 ↑ proteasome maturation protein ↑ immunoproteasome subunits ↑ LC3-II^+^ cytosolic structures resembling autophagosomes - Altered expression of cathepsins	B cells infected with EBV-related LCV were related to MS	^83^
Jagessar et al.^85^	Marmoset monkeys	- EAE model induced with rhMOG/CFA, MOG_34–56_/IFA - Anti-CD20 mAb anti-BLyS and anti-APRIL mAbs	- Anti-CD20 mAb reduced load of CalHV3 DNA in lymphoid organs - MOG_34–56_ group pulsed B lymphoblastic cell ↑ anti-MOG_34–56_ T cells and meningeal inflammation	Herpesvirus-transformed subset (CD20^+^ B cells) was related to EAE model	^85^
Axthelm et al.^86^	Japanese macaques	JME	- Gamma-herpesvirus was found in acute JME white matter lesions - Plaque-like demyelinated lesions	Association between MS and novel simian herpesvirus	^86^
Govindan et al.^87^	Japanese macaques	JME	↓ CD8^+^ T cell responses ↑ CD4^+^ Th1 and Th17 responses ↑ CD3^+^ T cells - Myelin antigens were identified	JME may shed new light on inflammatory demyelinating disease pathogenesis linked to gamma‐herpesvirus infection	^87^
Estep et al.^88^	Japanese macaques	Japanese macaque rhadinovirus (JMRV_17792_)	JMRV genome is similar to other gamma-herpesviruses	JMRV isolated from a Japanese macaque associated with inflammatory demyelinating encephalomyelitis (MS in humans)	^88^

Several key studies have used NHP models to investigate the pathogenesis of MS and the role of EBV or EBV-like viruses in this process. Haanstra et al. utilized rhesus macaques (*Macaca mulatta*) to study the effects of herpesvirus papio in combination with an encephalitogenic self-peptide (MOG_34–56_) and a capsid protein of cytomegalovirus (CMVmcp_981–1003_). Their findings indicated an increase in CD3^+^CD8^+^ T cells, CD8^+^CD56^+^ T cells and CD3^−^CD56^+^ NK cells, alongside an increase in perivascular inflammatory lesions composed of CD3^+^ and CD68^+^ cells^82^. These results suggest that EBV-induced B-lymphoblastoid cells can cause encephalitis, highlighting a potential mechanism by which EBV may contribute to MS pathology. Jagessar et al. examined LCV infection in NHP B cells and found increased expression of CD70, CD80, proteasome maturation protein, immunoproteasome subunits and LC3-II^+^ cytosolic structures resembling autophagosomes, along with altered cathepsin expression^83^. These changes suggest that B cells infected with EBV-related LCV could be linked to MS, highlighting the virus’s potential role in altering immune responses. Anwar Jagessar et al. used marmoset monkeys in an EAE model induced with rhMOG/CFA or MOG_34–56_/IFA. Treatments included anti-CD20 monoclonal antibodies (mAb) and antibodies targeting BLyS and APRIL. CalHV3 is a type of γ-herpesvirus found in marmosets. It has numerous functional similarities with EBV, such as the ability to transform B cells^84^. The anti-CD20 mAb reduced the load of CalHV3 DNA in lymphoid organs, while the MOG_34–56_ group showed increased anti-MOG_34–56_ T cells and meningeal inflammation^85^. This study suggests that a herpesvirus-transformed subset of CD20^+^ B cells may be relevant to the EAE model, providing a potential link to MS. Axthelm et al. studied Japanese macaques (*Macaca fuscata*) with a spontaneously occurring MS-like disease called Japanese macaque encephalomyelitis (JME). They isolated a novel gamma-herpesvirus in acute JME white matter lesions and plaque-like demyelinated lesions^86^. Their results support an association between MS and a novel simian herpesvirus, reinforcing the hypothesis that viral infections may play a role in MS pathogenesis. Govindan et al. further explored JME in Japanese macaques, noting decreased CD8^+^ T cell responses, increased CD4 Th1 and Th17 responses, and increased CD3^+^ T cells^87^. They also identified myelin antigens, suggesting that JME could provide new insights into the pathogenesis of inflammatory demyelinating diseases linked to gamma-herpesvirus infection. Estep et al. identified Japanese macaque rhadinovirus (JMRV17792), whose genome is similar to other gamma-herpesviruses^88^. They isolated JMRV from a Japanese macaque with inflammatory demyelinating encephalomyelitis, akin to MS in humans. This finding underscores the potential of using NHP models to understand the viral contributions to MS.

While NHP models have advanced our understanding of EBV and MS, the role of CD8^+^ T cell deficiency, particularly with aging, remains underexplored. Current studies focus on broader immunopathological mechanisms, but the impact of age-related CD8^+^ T cell decline on MS progression is unclear^8^. Aging reduces CD8^+^ T cell numbers and function, impairing EBV control and potentially exacerbating MS, but no studies have examined CD8^+^ T cell deficiency in aged NHP models (Table 1). Longitudinal studies tracking CD8^+^ T cells, EBV load and MS-like symptoms in aging NHPs are needed to clarify this relationship and explore therapeutic strategies to enhance CD8^+^ T cell function in older patients.

### CMV and its potential protective effect in MS

While EBV is linked to MS pathogenesis, cytomegalovirus (CMV) may play a protective role by enhancing CD8^+^ T cell responses. CMV infection increases CMV-specific CD8^+^ T cells, which may also bolster immunity against EBV, potentially reducing its reactivation and mitigating MS-related pathology. A study by Bjornevik et al. (2021) showed that CMV infection enhances the overall repertoire of CD8^+^ T cells that are cross-reactive to EBV, which may contribute to the suppression of EBV-driven immune dysregulation in MS^4^. These findings imply that CMV infection could potentially mitigate the autoimmune processes associated with MS. In addition, CMV infection has been shown to lead to an expansion of the CMV-specific CD8^+^ T cell pool, which might be beneficial in maintaining immune balance. This cross-reactivity between CMV and EBV-specific CD8^+^ T cells could reduce EBV-induced pathology, acting as a protective mechanism in patients with MS^89^.

## Considerations for developing novel EBV-induced NHP models of MS

NHP models offer great advantages for studying the role of EBV in MS due to their close physiological and immunological resemblance to humans. To develop and refine these models effectively, it is crucial to incorporate specific risk factors known to influence EBV infection and MS susceptibility. This section outlines strategies for creating robust NHP models to elucidate the contribution of EBV to the pathogenesis of MS development.

### CD8^+^ T cell depletion

CD8^+^ T cells are critical for controlling viral infections, including EBV^44^, but the specific effects of CD8^+^ T cell depletion on EBV infection and MS pathogenesis in NHP models are not well explored. Experimental depletion of CD8^+^ T cells in NHP models could help to elucidate their role in controlling EBV infection by assessing how the absence of CD8^+^ T cells affects viral load, persistence and reactivation. Previous data suggest that CD8^+^ T cell depletion leads to increased viral replication and dissemination, highlighting their importance in maintaining viral latency^64^. The role of CD8^+^ T cells in preventing autoimmune responses in the context of EBV infection is critical. Depletion studies could investigate whether the absence of CD8^+^ T cells exacerbates neuroinflammation and demyelination, potentially mimicking MS-like pathology. Understanding how CD8^+^ T cells modulate immune responses to myelin antigens in the presence of EBV-infected B cells is crucial. While NHP models have been instrumental in revealing the intricate relationship between EBV and MS, the role of CD8^+^ T cell deficiency in this dynamic, particularly in the context of aging, remains underinvestigated. Filling this research gap will allow a comprehensive understanding of MS and inform effective therapeutic strategies that address both viral control and age-related immune decline.

### Selecting nonprotective Mamu haplotypes

In rhesus macaques, the Mamu MHC class I and II alleles exhibit functional similarities to human HLA molecules, making them an important factor in the development of EBV-induced MS models. Specific Mamu haplotypes influence the immune response to viral infections, particularly the ability of CD8^+^ T cells to recognize and eliminate EBV-infected cells, which is a key determinant in controlling viral persistence and immune dysregulation. Identifying and selecting NHPs with Mamu haplotypes that mirror MS-associated HLA risk alleles is essential for model optimization. Previous studies have shown that HLA-A*02 and HLA-B*07 are associated with differential immune responses to viral infections, including EBV and CD8 response^90,91^. Given that HLA class II alleles are critical for antigen presentation and shaping adaptive immune responses, targeting Mamu haplotypes analogous to MS-associated HLA variants would help to establish a more translationally relevant model of EBV-induced MS. Future studies should genotype NHPs for Mamu haplotypes before infection to ensure appropriate selection. Integrating transcriptomic and proteomic analyses can further elucidate genetic influences on EBV-induced neuroinflammation. Refining NHP models based on Mamu haplotypes will improve reproducibility and translational relevance, aiding the development of targeted immunotherapies for MS.

### Vitamin D deficiency

The risk of MS has been linked to low vitamin D levels before the onset of the disease, and EBV infection is considered a necessary factor for MS development^92^. Vitamin D receptors are present on EBV-infected B cells, antigen-presenting cells and activated lymphocytes. The bioactive metabolite of vitamin D, dihydroxyvitamin D3, suppresses antibody production and T cell proliferation and promotes a shift in T cells toward a less harmful Th2 phenotype^93^. It has been suggested that reduced sunlight and vitamin D levels at higher latitudes may contribute to the development of autoimmune diseases by exacerbating CD8^+^ T cell deficiency, which, in turn, impairs the control of EBV^94^. Considering the role of vitamin D in regulating immune responses and controlling EBV infection, inducing vitamin D deficiency in NHP models can offer insights into its relationship with MS^17^. Vitamin D deficiency in NHPs can be induced with a diet devoid of vitamin D2 and D3 but with adequate levels of other nutrients to prevent confounding deficiencies. Regular monitoring of serum 25(OH)D levels and immune markers, including CD8^+^ T cell counts and function, ensures controlled vitamin D deficiency, allowing studies on its impact on EBV control, immune dysregulation and MS development.

### Selecting female monkeys

Women are approximately three times more likely than men to develop MS^95^. Given the established sex-specific differences, focusing on female NHPs in model development is crucial. Although NHP models can serve as a critical link between rodent EAE models and human patients with MS^96,97^, female primates would be more frequently afflicted with MS, and using female models can help to reveal sex-specific mechanisms of disease onset and progression. By studying these models, future studies can gain insights into how hormonal and genetic factors interact with EBV to influence MS development.

### Selecting susceptible ages of NHPs

Age at EBV infection is crucial for understanding its association with MS. In humans, the strongest correlation between EBV and MS is observed among individuals infected between the ages of 14 and 20 years. Most MS diagnoses occur at age 20–50, often many years after initial EBV exposure^3^. To accurately translate this in rhesus macaques, it would be pertinent to focus on young adult females aged 4–6 years. An unpublished study noted that no demyelinating lesions were found in rhesus macaques infected with LCV, suggesting that the absence of lesions could be due to the fact that the subjects were predominantly males and infants or juveniles, who are too young to exhibit such lesions.

## Immunotherapies in MS

EBV-targeted immunotherapies are being explored to reverse exhausted EBV-targeted T cell responses, particularly the CD8^+^ cytotoxic T lymphocyte (CTL) response, which is thought to lead to poor virus control and increased MS disease activity^54,98^. For example, the adoptive transfer of ex-vivo-expanded autologous EBV-specific CD8^+^ T cells is directed against viral latent proteins^98^. In an open-label trial of patients with progressive MS, escalating doses of ex-vivo-expanded autologous EBV-reactive T cells targeting EBNA1, LMP1 and LMP2A were administered, and seven out of ten showed improvement^99^. This approach was subsequently explored using partially MHC-matched allogenic EBV-specific T cells (clinicaltrials.gov NCT03283826)^100^; however, the phase 2 exploratory trial was negative^101^. Whether this trial failed because of the poor survival of these allogeneic cells is unclear^102^. Another possible explanation for the results is inadequate trial design, which included patients with MS with high levels of disability^102^. Another approach being explored is therapeutic EBV vaccination to boost T cell responses to EBV^103^, ideally in NHP models of MS.

## Conclusion

The relationship between EBV and MS represents a complex interaction of viral, genetic and environmental factors. This Review highlights the critical need for developing and refining NHP models to address clinically relevant questions about the role of EBV in MS pathogenesis. By focusing on key EBV-related risk factors, such as infectious mononucleosis, timing of infection, genetic susceptibility (HLA haplotypes), sex-specific differences, vitamin D levels and CD8^+^ T cell deficiency, we can better understand how EBV may trigger or drive MS. The proposed NHP-EBV infection models, especially those incorporating CD8 depletion and other relevant factors, offer a promising approach to determining whether EBV acts as a trigger for MS or a driver of its progression. Such models will not only facilitate the evaluation of these theories but also help to reveal why most EBV-infected individuals do not develop MS. These insights could inform the development of disease-modifying therapies, including EBV-targeted immunotherapies and preventive vaccines. By bridging gaps in our understanding of MS and EBV, we can translate research into effective strategies for managing and preventing MS. The integration of these models into clinical research could lead to major advancements in treatment and prevention, ultimately improving outcomes for individuals at risk of or living with MS (Boxes 1 and 2).

Box 1 Testing theories about EBV and MS on NHP models EBV is thought to serve as a trigger for MS onset. NHP models infected with EBV will allow future studies to investigate this theory by observing whether the introduction of EBV precedes the development of MS-like symptoms. This approach can help to determine if EBV acts as an initial catalyst for disease onset. Another hypothesis is that EBV functions as a driver of MS progression. NHP models can be used to investigate whether chronic EBV infection exacerbates MS symptoms and accelerates disease progression. By studying the long-term effects of EBV on MS development, researchers can assess the role of EBV in driving disease progression.

Box 2 Addressing the gaps in understanding why a vast majority of EBV-infected individuals do not develop MS Despite the widespread prevalence of EBV infection, many individuals do not develop MS^6^, so a key goal is to understand the underlying factors that protect some individuals from MS despite EBV exposure. NHP models can be used to explore these protective factors by comparing EBV-infected primates that do and do not develop MS-like symptoms. This comparison can help to identify potential genetic, immunological or environmental factors that contribute to MS resistance. Based on insights from NHP models, this Review proposes several potential disease-modifying therapeutic options and vaccine strategies. By identifying key factors that influence EBV-induced MS, future research can inform the development of targeted therapies aimed at modifying disease progression or preventing MS. For example, immunomodulatory therapies could be developed to enhance CD8^+^ T cell function or modulate immune responses to EBV. Gene therapy may offer a way to correct genetic predispositions associated with MS, and investigating the efficacy of vitamin D supplementation in the context of EBV infection could reveal its potential role in preventing or treating MS. Developing vaccines targeting EBV or its interactions with host immune cells may also help to prevent MS onset. By integrating these approaches, researchers can advance our understanding of MS pathogenesis and create effective strategies for managing and preventing this debilitating disease.

## Data sharing statement

Data will be made available on request.
